# Infection with influenza A(H1N1)pdm09 during the first wave of the 2009 pandemic: Evidence from a longitudinal seroepidemiologic study in Dhaka, Bangladesh

**DOI:** 10.1111/irv.12462

**Published:** 2017-07-26

**Authors:** Sharifa Nasreen, Mustafizur Rahman, Kathy Hancock, Jacqueline M. Katz, Doli Goswami, Katharine Sturm‐Ramirez, Crystal Holiday, Stacie Jefferson, Alicia Branch, David Wang, Vic Veguilla, Marc‐Alain Widdowson, Alicia M. Fry, W. Abdullah Brooks

**Affiliations:** ^1^ icddr,b Dhaka Bangladesh; ^2^ Influenza Division Centers for Disease Control and Prevention (CDC) Atlanta GA USA; ^3^ Johns Hopkins University Baltimore MD USA

**Keywords:** Bangladesh, H1N1 subtype, influenza A virus, pandemic, seroconversion, seroepidemiologic studies

## Abstract

**Background:**

We determined influenza A(H1N1)pdm09 antibody levels before and after the first wave of the pandemic in an urban community in Dhaka, Bangladesh.

**Methods:**

We identified a cohort of households by stratified random sampling. We collected baseline serum specimens during July‐August 2009, just prior to the initial wave of the 2009 pandemic in this community and a second specimen during November 2009, after the pandemic peak. Paired sera were tested for antibodies against A(H1N1)pdm09 virus using microneutralization assay and hemagglutinin inhibition (HI) assay. A fourfold increase in antibody titer by either assay with a titer of ≥40 in the convalescent sera was considered a seroconversion. At baseline, an HI titer of ≥40 was considered seropositive. We collected information on clinical illness from weekly home visits.

**Results:**

We tested 779 paired sera from the participants. At baseline, before the pandemic wave, 1% overall and 3% of persons >60 years old were seropositive. After the first wave of the pandemic, 211 (27%) individuals seroconverted against A(H1N1)pdm09. Children aged 5‐17 years had the highest proportion (37%) of seroconversion. Among 264 (34%) persons with information on clinical illness, 191 (72%) had illness >3 weeks prior to collection of the follow‐up sera and 73 (38%) seroconverted. Sixteen (22%) of these 73 seroconverted participants reported no clinical illness.

**Conclusion:**

After the first pandemic wave in Dhaka, one in four persons were infected by A(H1N1)pdm09 virus and the highest burden of infection was among the school‐aged children. Seroprevalence studies supplement traditional surveillance systems to estimate infection burden.

## 1 BACKGROUND

The first laboratory‐confirmed case of influenza A(H1N1)pdm09 was identified in the United States in April 2009.^1^ The virus rapidly spread globally leading to the World Health Organization declaring an influenza pandemic on 11 June 2009.^2^ In Bangladesh, the first case of influenza A(H1N1)pdm09 virus infection was detected on 18 June 2009 through an event‐based surveillance program^3^; national hospital‐based influenza surveillance started detecting A(H1N1)pdm09 cases in August 2009, indicating spread of the virus to the general population.^4^ Overall, different surveillance platforms identified 1371 laboratory‐confirmed cases and 28 deaths during June 2009‐October 2010 in Bangladesh.^5^ However, this number is an underestimate of the burden of A(H1N1)pdm09 infections in Bangladesh because not all ill people seek health care and the number of surveillance hospitals is limited.

Longitudinal cohort studies measuring the change in antibody titers over time are an important adjunct to surveillance and assist in the estimation of the true infection burden. We followed a community cohort in a low‐income urban area of Dhaka, Bangladesh, from the beginning of the pandemic in Bangladesh until after the first wave of illness. In this study, we report A(H1N1)pdm09 antibody levels before and after the first wave of the pandemic following the Reporting Of Seroepidemiologic Studies for Influenza (ROSES‐I) guidelines provided by the Consortium for the Standardization of Influenza Seroepidemiology (CONCISE).^6^ These data provide insight into the burden of A(H1N1)pdm09 infections, including clinical and subclinical infection, and residual susceptibility after the first wave of the pandemic in Dhaka, Bangladesh.

## 2 METHODS

### 2.1 Ethics statement

Informed consent was obtained from all adults. For children aged 8‐17 years, consent was obtained from both the child and their parents or guardians, while informed consent was obtained from the parents or guardians for children aged <8 years. The study was approved by both the institutional review boards at icddr,b and the U.S. Centers for Disease Control and Prevention (CDC).

### 2.2 Setting

This was a nested study within a cohort of households under surveillance as part of a double‐blind randomized controlled clinical efficacy trial of oseltamivir among individuals ≥1 year old in Kamalapur, Dhaka, from May 2008 to December 2010.^7^ For the main study, 6600 households selected by stratified cluster randomization were visited weekly by trained field research assistants who screened for clinical illness using standardized questionnaires, as previously described.^8^

### 2.3 Serum specimen collection for the influenza A(H1N1)pdm09 sero study

We approached a 10% sample of the households enrolled in surveillance for the oseltamivir study using stratified random selection. We estimated that this subset would include ~700 households or 3000 individuals, a sample size adequate to detect a 10% cumulative incidence of infection among persons. We collected baseline serum specimens during 29 July‐18 August 2009, just prior to the initial wave of the 2009 pandemic in this community. Follow‐up serum specimens were collected between 4 and 25 November, 2009 from 3048 individuals, after the first pandemic wave in this community.

### 2.4 Laboratory analysis

We randomly selected ~20% of the paired sera from participants aged <60 years, while all sera from participants aged >60 years (N=71) were tested. We chose to include these sera samples in our study instead of the paired sera collected from 3048 individuals considering the resources that would be required for the laboratory analysis of all the samples. Random selection of the samples for laboratory testing likely did not introduce any selection bias because the study participants were similar to the overall surveillance population with respect to age and sex distribution (Table 1). Paired sera were tested by microneutralization (MN) and 0.5% turkey red blood cell hemagglutination‐inhibition (HI) assays using A/Mexico/4108/2009, an A/California/07/2009‐like virus. Seroconversion was defined as a fourfold rise in antibody titer by either assay and required a minimum titer of 40 in the convalescent serum sample. At baseline, an HI titer of ≥40 was considered seropositive. We present baseline titers of ≥20 and ≥40 by HI assay because sera of ten participants (~1% of total sera) having a titer of ≥20 by HI assay were not tested by MN assay due to insufficient volume, and thus, an HI titer of ≥20 would be sufficiently sensitive in detecting infection with A(H1N1)pdm09 among the participants aged <60 years.^9^ Furthermore, in a previous study, 11% of confirmed cases in England did not have HI titer of ≥32 at baseline suggesting that a cutoff HI titer value of ≥40 could underestimate infection.^10^ We also present baseline serology of both HI titer of ≥20 and MN titer of ≥40 because this combined titer provided the best balance of sensitivity and specificity for US individuals aged <60 years.^9^

**Table 1:** Characteristics of the study participants and the overall surveillance population, Kamalapur, Bangladesh, 2009

Age group	Study participants (N=780)		Kamalapur (DSS) surveillance population (N=114 146)	
	n (%)^a^	Male, n (%)^b^	n (%)^a^	Male, n (%)^b^
<5 y	107 (14)	59 (55)	18 208 (16)	9187 (50)
5‐17 y	217 (28)	129 (59)	27 995 (25)	13 993 (50)
18‐39 y	289 (37)	105 (36)	47 201 (41)	22 301 (47)
40‐59 y	96 (12)	45 (47)	17 008 (15)	9549 (56)
>60 y	71 (9)	42 (59)	3734 (3)	2178 (58)

a Column percent.

b Row percent.

### 2.5 Clinical data of participants with serology results

Information on clinical illness was collected through weekly home visit questionnaires and project clinic visit documents (case report form and clinic visit sheets) for the participants with serology results, as previously described.^8^

### 2.6 Data analysis

We calculated 95% CI for the proportions of baseline serology (titers of ≥20 and ≥40 by HI assay and a titer of ≥40 by MN assay) and proportions of seroconversion against A(H1N1)pdm09 virus antibodies using binomial distribution. We adjusted the proportion of seroconversions (by HI and MN assay) for household clustering using clustered sandwich estimator for variance‐covariance matrix estimation. We considered symptomatic A(H1N1)pdm09 infection among the seroconverted participants if the symptoms developed 3 weeks or more prior to the collection of follow‐up serum specimen. We estimated A(H1N1)pdm09 infection among Dhaka district population by applying the overall proportion of adjusted seroconversion observed among our study participants.

## 3 RESULTS

Among 6600 households under surveillance for the drug study, 930 households were approached and asked to consent to the serology study; 220 members from 44 households refused to participate. We collected baseline serum specimens from 3647 individuals; 116 individuals refused to give a serum sample. We collected follow‐up serum specimens from 3048 individuals; 198 participants refused to provide follow‐up serum.

We tested 709 randomly selected paired sera from participants aged <60 years and 71 paired sera from participants aged >60 years. Overall, 779/780 provided paired sera; one participant provided only a single serum at baseline. The age range of the participants was one month to 107 years. The highest proportion of participants was aged 18‐39 years (Table 1). None of the participants had ever received any influenza vaccine.

### 3.1 Serology against A(H1N1)pdm09 virus

At baseline, nine (1%) of 779 participants were seropositive. A similar proportion of participants had A(H1N1)pdm09 virus antibody titers ≥20 by HI assay and ≥40 by MN assay (Table 2). The highest proportion was among participants aged >60 years. The median time interval between collection of baseline and follow‐up sera sample was 98 days (interquartile range, IQR: 93‐98 days). Among the household participants providing paired sera, 211/779 (27%) seroconverted against A(H1N1)pdm09 virus. Children aged 5‐17 years had the highest proportion of seroconversion against A(H1N1)pdm09 virus (37% by either assay) (Table 3).

**Table 2:** Baseline (pre‐ or very early pandemic) antibody titers against A(H1N1)pdm09 virus among the participants, Kamalapur, Bangladesh, 2009

Age groups (years)	n/N (%, 95% CI)			
	HI assay titer		MN assay titer	HI assay titer ≥20 AND MN assay titer ≥40
	≥20	≥40	≥40	
<5	0/107 (0, 0‐3)	0/107 (0, 0‐3)	0/104 (0, 0‐3)	0/104 (0, 0‐3)
5‐17	4/216 (2, 1‐5)	3/216 (1, 0‐4)	2/208 (1, 0‐3)	1/207 (0, 0‐3)
18‐39	12/289 (4, 2‐7)	3/289 (1, 0‐3)	11/263 (4, 2‐7)	4/263 (2, 0‐4)
40‐59	4/96 (4, 1‐10)	1/96 (1, 0‐6)	3/89 (3, 1‐10)	2/89 (2, 0‐8)
>60	5/71 (7, 2‐16)	2/71 (3, 0‐10)	6/71 (8, 3‐17)	3/71 (4, 1‐12)
All age group	25/779 (3, 2‐5)	9/779 (1, 1‐2)	22/735 (3, 2‐4)	10/734 (1, 1‐2)

**Table 3:** Seroconversion against A(H1N1)pdm09 virus after the first peak of pandemic, Kamalapur, Bangladesh, 2009

Age groups (years)	Seroconversion,^a^ n/N (%, 95% CI)			%, 95% CI adjusted for household clustering
	HI assay	MN assay	HI or MN assay	HI or MN assay
<5	19/107 (18, 11‐26)	26/104 (25, 17‐34)	26/107 (24, 17‐34)	24 (16‐33)
5‐17	54/216 (25, 19‐31)	78/208 (38, 31‐44)	80/217 (37, 30‐44)	37 (30‐44)
18‐39	36/289 (12, 9‐17)	65/263 (25, 20‐30)	65/289 (22, 18‐28)	22 (17‐28)
40‐59	19/96 (20, 12‐29)	25/89 (28, 19‐39)	26/96 (27, 19‐37)	27 (18‐37)
>60	11/70 (16, 8‐26)	13/70 (19, 10‐30)	14/70 (20, 11‐31)	20 (10‐30)
All age group	139/778 (18, 15‐21)	207/734 (28, 25‐32)	211/779 (27, 24‐30)	27 (23‐31)

a Seroconversion was defined as a fourfold rise in antibody titer by hemagglutination‐inhibition (HI) or microneutralization (MN) assay with a minimum titer of 40 in the convalescent serum sample.

### 3.2 Clinical profile of the participants

Clinical illness information from weekly household visits was available for 739 participants. However, 264 of 780 (34%) individuals with serology results had information on illness >3 weeks prior to collection of follow‐up sera. Overall, 72% (191/264) of these participants reported a febrile or respiratory illness >3 weeks prior to collection of follow‐up sera (Table 4). Among those with clinical illness, 73 (38%; range 31%‐46% among age groups) seroconverted against A(H1N1)pdm09 virus. Among the 73 participants without a report of clinical illness, 16 (22%) seroconverted against A(H1N1)pdm09 virus. Children aged <5 years comprised 9 (56.2%) of 16 people with asymptomatic infections.

**Table 4:** Clinical profile and seroconversion compatible with A(H1N1)pdm09 infection (clinical illness information >3 wk prior to collection of follow‐up sera) Kamalapur, Bangladesh 2009

Age groups (years)	Had Clinical illness (N=191)			No clinical illness (N=73)		
	Total	Seroconversion		Total	Seroconversion	
		n	%		n	%
<5 y (n=77)	36	11	30.6	41	9	22.0
5‐17 (n=56)	52	24	46.2	4	1	25.0
18‐39 (n=84)	72	27	37.5	12	1	8.3
40‐59 (n=28)	19	7	36.8	9	4	44.4
>60 (n=19)	12	4	33.3	7	1	14.3
All age groups (n=264)	191	73	38.2	73	16	21.9

### 3.3 Estimated A(H1N1)pdm09 infection among Dhaka population

Applying the overall adjusted proportion of seroconversion found among our study participants to the population of Dhaka, we estimated that 3 251 874 (27% of 12 043 977) people in Dhaka city were infected by A(H1N1)pdm09 during the first wave of pandemic in 2009.

## 4 DISCUSSION

Our study findings suggest that one in four immune‐naïve persons in Bangladesh was infected by A(H1N1)pdm09 virus during the first wave of pandemic influenza in 2009. This proportion is within the range reported from Malaysia (18.2%‐26.0%), Australia (25.0%‐31.7%) 11, 12 and the overall cumulative incidence (20%‐27%) in 19 countries reported by a recent meta‐analysis,^13^ but higher than the rates in Singapore (13%) and Hong Kong (14%) 14, 15 and lower than the USA (35%).^16^ The highest proportion of A(H1N1)pdm09 virus infection occurred among school‐aged children in our study, consistent with findings from these other studies. Older adults aged >40 had more infections than young adults or children <5 years. We found serologic evidence for infection among ~20% of those without symptoms; however, most of these occurred among children aged <5 years. These data help to understand the full burden of H1N1pdm09 infections in Bangladesh after the first wave of the pandemic.

Interestingly, we found a very low level of immunity to A(H1N1)pdm09 prior to the first wave of the pandemic, even among older adults. Even after evaluating different titer thresholds for a seropositivity definition (HI titer ≥20 or ≥40, or combined HI titer ≥20 and MN titer ≥40), the proportion of older adults with seropositivity at baseline was low but within the range reported from China.^17^ It was substantially lower than in estimates of pre‐pandemic UK, USA, and Australian populations.^10^, ^18^, ^19^

Our study is unique in that we could follow a cohort longitudinally. Most seroprevalence studies from the pandemic were cross‐sectional and included different study populations in pre‐ and post‐first pandemic wave samples.^20^ Other strengths of our study were that we included all age groups. To our knowledge, no other published study has reported on all age groups at the start of the pandemic. Importantly, we also systematically collected clinical information on a subset of the surveyed population. Clinical data are generally not available when serosurveys use blood bank samples or residual laboratory sera.^20^

There are several important limitations to our study. Although we started our study before A(H1N1)pdm09 virus spread to the general population in the country, it is possible that some of the baseline titers were due to recent A(H1N1)pdm09 infections among our study population; however, at the time of launching our survey, our ongoing surveillance systems had not detected such cases. The survey was conducted in a single field site, and infection rates may have differed in other parts of the city and country; however, comparisons of influenza circulation in the site to those from the hospital network, which is nationwide, suggest that circulation patterns in this community are comparable.^8^, ^21^ We may have missed some infections by relying only on serology. This could have disproportionately affected the very young and the elderly, resulting in an underestimation of infection in those age groups. Despite these limitations, the findings from this study provide insight into the propagation of a novel virus through a densely populated but immunologically naive population with a high background rate of seasonal influenza infection.

## 5 CONCLUSION

Our study demonstrated that most of the study participants were susceptible to A(H1N1)pdm09 infection before the first wave of the pandemic and that even the majority of the elderly population lacked protective immunity from previous exposure to similar virus. About a quarter of the population was infected and the highest detected burden of infection occurred among the school‐aged children during the first wave of the pandemic. Three‐fourths of the population remained uninfected and susceptible at the end of the first pandemic wave.
